# Improving kidney transplantation through the addition of pharmacological agents to hypothermic preservation solutions: a scoping review

**DOI:** 10.3389/fphar.2025.1692398

**Published:** 2026-01-23

**Authors:** Vincent Mayoral, Tom Darius, Sarah Bruneau, Christophe Masset, Jérôme Rigaud, Gilles Blancho, Thomas Prudhomme, Julien Branchereau, Benoît Mesnard

**Affiliations:** 1 Center for Research in Transplantation and Translational Immunology, Nantes Université, CHU Nantes, INSERM, UMR 1064, Nantes, France; 2 Department of Urology, Charles Nicolle Hospital, University of Rouen Normandy, Rouen, France; 3 Institut de Recherche Experimentale et Clinique (IREC), Université Catholique de Louvain, Brussels, Belgium; 4 Surgery and Abdominal Transplant Unit, Cliniques Universitaires Saint-Luc, Université Catholique de Louvain, Brussels, Belgium; 5 Department of Urology and Transplantation Surgery, Nantes University Hospital, Nantes, France

**Keywords:** kidney transplantation, organ preservation, drug therapy, tissue donor, reperfusion injury

## Abstract

The increasing use of extended kidney grafts to bridge organ shortage has led to delayed and impaired graft function, warranting the development of new preservation strategies. In addition to hypothermic machine perfusion, the addition of pharmacological agents to preservation solutions has been primarily investigated, with a few promising agents making their way into clinical trials. This review aimed to identify and summarize current literature studies on pharmacological treatment additives for hypothermic kidney graft preservation. A scoping review was conducted according to the Preferred Reporting Items for Systematic Reviews and Meta-Analyses extension for Scoping Reviews (PRISMA-ScR) guidelines. A comprehensive literature search was performed using Medline and Cochrane Library databases until 1 December 2023. All studies published in English reporting on pharmacological supplementation of preservation solutions to improve hypothermic kidney graft preservation were included. A total of 67 records were retrieved, all of which were preclinical except one. Of these, 8 were conducted on cellular models, 21 on *ex vivo* kidneys, and 38 on animal kidney transplantations. A total of 40 pharmacological agents were evaluated based on the key markers of ischemia–reperfusion injury (IRI) pathophysiology, most of them showing promise in kidney preservation. Although promising, with numerous preclinical studies identifying various effective additives, the pharmacological treatment additive strategy to improve hypothermic kidney preservation has still not been translated into clinical practice. Clinical investigations should be promoted to support few ongoing trials offering encouraging outcomes.

## 1 Introduction

Kidney transplantation is the gold standard for the treatment of end-stage renal disease, with living donor kidneys yielding superior long-term outcomes (Lentine et al., 2024). To cope with increasing demands and limited graft availability, the use of expanded criteria donors (ECDs) and donors after circulatory death (DCD) is increasing, further extending the limits of donor eligibility criteria (Lomero et al., 2020). These extended kidney grafts are prone to ischemia–reperfusion injuries (IRIs), resulting in an increased incidence of delayed graft function (DGF) and its related costs and recipient morbidity (Barreda Monteoliva et al., 2022). Improving the protection of these vulnerable organs would be beneficial to optimize the limited supply of kidney grafts. Many strategies have been investigated to reduce IRI and improve graft and patient survival (Moers et al., 2012; Mesnard et al., 2022; Branchereau et al., 2022). Hypothermic machine perfusion (HMP) has already been demonstrated to be superior to static cold storage (SCS) in decreasing DGF in DCD and ECD kidneys, especially when applied as a continuous preservation strategy, and has been implemented worldwide in clinical practice for decades (Tingle et al., 2024). Pharmacological treatment additives are another key strategy for improving the efficacy of organ preservation solutions and have been increasingly investigated in recent years using multiple agents with distinct mechanisms of action. Nevertheless, despite these efforts, no clinical implementation has been achieved yet. Considering the wide spectrum of agents and strategies reported to date, the aim of this review was to summarize the nature and extent of research on pharmacologically improved hypothermic kidney preservation.

## 2 Methods

A scoping review was conducted and reported according to the Preferred Reporting Items for Systematic Reviews and Meta-Analyses extension for Scoping Reviews (PRISMA-ScR) guidelines. The primary objective of the report was to retrieve all published data addressing the improvement of hypothermic kidney preservation for transplantation through the addition of pharmacological agents to preservation solutions. The review protocol had been registered at the Open Science Framework DOI 10.17605/OSF.IO/3VA4X and can be accessed at https://archive.org/details/osf-registrations-3va4x-v1.

Two authors (VM and BM) agreed on the key words to conduct the literature search. Searches were performed in the Medline and the Cochrane Library as [(“Kidney Transplantation” [Mesh]) AND “Organ Preservation Solutions/pharmacology” [Mesh]] and (“Kidney transplantation” [Title/Abstract/Keyword] AND “Preservation” [Title/Abstract/Keyword]), respectively. Databases were accessed between 11 November 2023 and 1 December 2023. Relevant quoted articles in the reference list of already identified articles were also included. Article selection and removal of duplicates were managed using Zotero 6.0.36. Scanning of titles and abstracts was performed by two authors (VM and BM), and full-text reading for eligibility was conducted by the first author. Final decision for inclusion was based on consensus of both authors. In case of discrepancies, a third author resolved concerns regarding eligibility (JB). All published articles written in English until 1 December 2023 reporting on the use of a pharmacologically supplemented preservation solution to reduce IRI in any model of hypothermic kidney preservation, from cellular preclinical investigations to clinical studies, were included. Exclusion criteria included the lack of pharmacological additives to standard organ preservation fluids, absence of a clear statement regarding pharmacological additive usage, or no use of a preservation solution. Abstract-only articles, reviews, and unpublished data were also excluded. Oxygen supplementation and its transporters, along with vasodilators without specific activity against IRI, were not covered under the scope of this review.

Study characteristics and results were then charted for the following information: first author and year of publication, country where the study was conducted, type of study (preclinical or clinical), investigated pharmacology, characteristics of the hypothermic preservation model (individuals studied and preservation conditions), pharmacology doses, and results obtained. All the collected data were exported into an Excel sheet by the first author and then reviewed and agreed by all the authors. The results were presented for their general characteristics and outcomes, before being detailed one by one for relevant data from clinical to preclinical studies, grouped by pharmacology family and mechanism of action.

## 3 Results

### 3.1 Charting process and data summary

**FIGURE 1:**
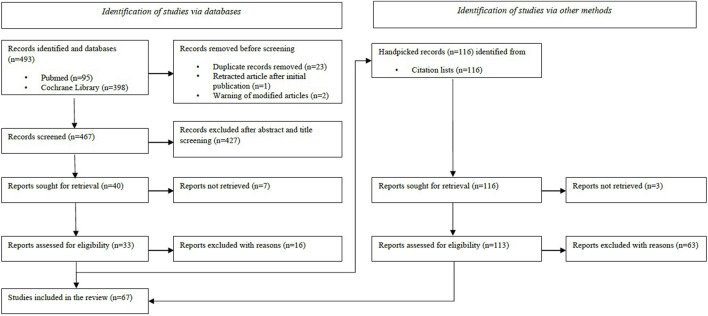
Flow chart diagram.

The literature search found 609 articles, of which 493 were identified from databases and 116 were collected from the citation list of the included studies among these 493 articles. After removal of duplicates and retracted articles, 583 were screened for title and abstract, with 146 selected for full-text review. After exclusion of 79 ineligible articles not focused on preservation solution supplementation in a kidney transplantation model, the charting process led to the inclusion of 67 articles (Figure 1).

Of these 67 records, all, except three, were preclinical studies, 8 of which used a cellular-only-based model, 21 studied an *ex vivo* kidney model including 7 normothermic reperfusion strategies, and 38 performed animal kidney transplantation (whether allo- or syngeneic- or autotransplantation). Most of the studies were conducted using a porcine or murine model (whether isolated kindeys or transplantation model). The total number of pharmacological agents investigated in the selected studies was 40 (Table 1). The main purpose of the studies was to evaluate the roles of these agents in reducing renal IRI based on various outcomes as markers of oxidative stress (reactive oxygen species [ROS] levels, DNA damage, lipid peroxidation, and pro-/antioxidant enzyme activity), cell metabolism (ATP content, mitochondrial integrity and function, and lactate levels), inflammatory parameters (cytokine and chemokine levels and adhesion and prothrombotic molecule expression), cytoprotective signaling pathway activity (HO-1 and HIF1α), cell death (apoptosis and its effectors, LDH), kidney perfusion parameter kinetics, glomerular and tubular kidney function (glomerular filtration rate, proteinuria, urinary ion balance, and tubular damage urinary markers), histological kidney lesions (tubular necrosis, fibrosis, and immune cell infiltrate), kidney fibrosis scarring (epithelial–mesenchymal transformation effectors), transplanted animal survival, and primary non-function of the kidney.

**TABLE 1:** Included studies on pharmacological supplementation of hypothermic kidney preservation solutions.

Author, year of publication	Country	Type of study	Pharmacology	Studied individuals	Preservation conditions	Preservation duration	Pharmacology dose	Results
Nicholson et al. (1996)	United Kingdom	Clinical Phase III	Nicardipine	Human DBD	Flush + SCS Eurocollins	Clinical purpose	Nicardipin 2mg	No difference after W6 on: DFG, dialysis duration, time to creatinine drop, GFR, Acute rejection
Le Meur et al. (2020)	France	Clinical Phase I	M101	Human DBD	Flush + SCS or HMP UW French standard of care	Clinical purpose	1 g/L	No difference in adverse events at M3M12, enhanced recovery of creatininemia during D1-D7, no difference in DGF, ↘number of dialysis at M1
Sedigh et al. (2022)	Sweden	Clinical Phase I	Corline Heparin Conjugate (CHC)	Human DBD	HMP UW Swedish standard of care	Clinical purpose	CHC 100 mg (2 mg/mL) or placebo	No difference in adverse events at M1, no difference in DGF and dialysis, ↗blood loss during surgery, ↗time to creatininemia and cystatin C recovery, ↘GFR in the first week
Antioxydants: flavonoids								
Ahlenstiel et al. (2006)	Germany	Preclinical	Bioflavonoids	LLC-PK1	SCS UW/EC 4 °C	20h + 1h reperfusion	50-100 μM	In EC: ↗MTT-test, ↘LDH (quercetin, kaempferol), ↗partial MTT-test ↘LDH (fisetin, myricetin, morin) In UW: ↗partial MTT-test, ↘LDH (quercetin, kaempferol, luteolin, fisetin, myricetin, morin, catechin, silibinin) In both: ↘MDA levels (quercetin, kaempferol, luteolin)
Karhumäki et al. (2007)	Finland	Preclinical	Butylated Hydoxytoluene Quercetin Resveratrol Epigallocatechin	LLC-PK1	SCS UW 5 °C	16-18h + 24h reperfusion	0,05-30 μM	↘morphological alterations, ↘LDH, ↘ATP depletion, ↘ROS
Zhang et al. (2012)	China	Preclinical	Tanshinone IIA	Rat kidney	In situ Flush + SCS Celsior 4 °C	24/48h	100 μM	At 24/48h: ↘MDA, ↗SOD, ↘CHOP, ↘Casp12 cell+
Cassim et al. (2022)	France	Preclinical	ADD-10	LLC-PK1 pig autotransplant (DCD WIT 30min)	SCS UW 4 °C	24h	ADD-10 1%	↗cell survival, ↘morphological changes, ↗ATP preservation, ↘ROS, ↗basal respiration capacity ↘creatinemia and blood urea nitrogen (BUN) D7, ↘creatinine/urea peak duration and levels, ↘fibrosis
Soussi et al. (2019)	France	Preclinical	Vectisol	Primary human kidney endothelial cells Pig autotransplant Pig autotransplant (DCD WIT 1h)	SCS UW hypoxic 4 °C SCS Celsior/UW 4 °C HMP KPS 4 °C	24h + reperfusion 24h 23h	Unknown for cell model In vivo: 1,56 g/L	↗cell viability SCS: ↘creatininemia peak and recovery duration, ↘AUC FENa D1-14, ↘tubular cell detachment, ↘creatininemia and proteinuria at M1, ↘tubular atrophy and fibrosis HMP: ↘apoptosis, ↘Rox/DAPI, ↘AUC SOD activity D1-14, ↘AUC ASAT D1-14, ↘creatininemia and proteinuria at M3, ↘tubular atrophy and fibrosis at M3
Antioxydants: direct scavengers								
Killinger et al. (1992)	United States	Preclinical	Lazaroids	HUVEC	SCS Eurocolins 4 °C	48/96h + 4h reperfusion in culture medium	50 μM	↗MTT viability test at 48-96h (best U74500A with dose-dependent effect)
Salahudeen et al. (1999)	United States	Preclinical	2-Methyl AminoChroman Deferoxamine	LLC-PK1	SCS UW 4 °C	24/48/72h	2-MAC: 0,156 μM DFO: 250 μM	↘F2-isoprostanes with 2-MAC only
Salahudeen et al. (2000)	United States	Preclinical	2-Methyl AminoChroman Deferoxamine	Human renal proximal tubular cell	SCS UW 4 °C	24/48/72h	2-MAC: 0,156 or 1,56 μM DFO: 250 μM or 2,50 mM	↘LDH, ↘membrane lipid degradation, ↘GSH depletion (DFO only), ↘superoxide and hydrogen peroxide formation, ↘ATP depletion, ↘DNA damages, ↗cell proliferation preservation
Salahudeen et al. (2001)	United States	Preclinical	2-Methyl AminoChroman Deferoxamine	Human renal proximal tubular cell	SCS UW 4 °C	12/24/38/46h + 24h reperfusion in medium	2-MAC: 1,56 μM DFO: 2,50 mM	↘LDH, ↗preservation of mitochondrial and plasma membrane, ↗prevention of vacuolization and chromatin clumping, ↘apoptosis
Mitchell et al. (2011)	United States	Preclinical	Mitoquinone (MQ)	NRK-52E rat kidney proximal tubular cell Rat kidney	SCS UW/Viaspan 4 °C In situ saline Flush + SCS 4 °C	4h +/- 18h reperfusion in culture medium 4h	MQ: 1 μM or DecylTTP: 1 μM (= inactive MQ) MQ: 100 μM or DecylTTP: 100 μM	↘MitoSOX, ↘Nitrotyrosine, ↗mitochondrial respiratory chain activity, ↘cell death after reperfusion ↘MitoSOX and Nitrotyrosine on immunofuorescence, ↗mitochondrial respiratory chain activity, ↘tubular dilatation/brush border loss/cast formation, ↘apoptosis
Parajuli et al. (2012)	United States	Preclinical	Mitoquinone (MQ)	Pig kidney (ØWIT)	In situ Flush + SCS UW 4 °C	24/48h	100 μM	↘histology lesion score (brush border loss/epithelial detachment/cast formation) at 48h, ↘nitrotyrosine at 48h, ↗complex II+III activity at 24h only, ↘apoptosis at 48h
Verhoven et al. (2020)	United States	Preclinical	PrC-210	Rat kidney	In situ Flush UW 20 °C + SCS UW 4 °C	30h	0-30 mM + NaOH (UW pH adjustment)	↘caspase activity and ↘acute tubular necrosis (best at 20-30 mM), total protection from ROS lipid peroxidation and DNA breakage on isolated in vitro rat kidney cell mitochondria
McAnulty and Huang (1997)	United States	Preclinical	Vitamin C (VitC) Trolox (T) Deferoxamine (DFO)	Rabbit kidney cortex slices	Cortex slices 37 °C aerobic incubation + WIT + SCS UW 5 °C	30 min + 60 min + 18h + 3h30 reperfusion in warm physiologic buffer	VitC: 1 mM or DFO: 1 mM or T: 1 mM or VitC + DFO or VitC + T	At end-SCS: ↘MDA levels (T and DFO alone only), ↗lipid peroxides and conjugated dienes After reperfusion: ↘MDA levels (except VitC + DFO ↗), ↘Schiff bases (best DFO; and except for VitC and its combinations ↗)
McAnulty and Huang (1996)	United States	Preclinical	Vitamin C (VitC) Trolox (T)	Dog kidney (ØWIT)	Flush + SCS UW 2 °C	48h + 1h reperfusion of homogenate samples in 37° shaking water bath	T: 200 μM or VitC: 1 mM or T + VitC	↗conjugated dienes, ↗lipid peroxides, ↘uncoupler-stimulated respiration rates, no effect on ADP-stimulated respiration [negative impact]
Ostróżka-Cieślik et al. (2018)	Poland	Preclinical	Vitamin C (VitC) Prolactine (P)	Pig kidney	Flush + SCS Biolasol 4 °C	48h	VitC: 0,088 g/L P: 1 μg/L	No effect on AST/ALAT/LDH/Lactate
Ostróżka-Cieślik et al. (2021)	Poland	Preclinical	Zinc (Zn) Prolactine (P)	Pig kidney	Flush + SCS Biolasol 4 °C	2h before first perfusion and sampling + 48h end-perfusion and sampling	Zn: 1 μg/L or P: 0,1 μg/L or Zn + P	At 48h: ↗ALAT/ASAT/LDH and ↘Na+ and ↗K+ in perfusate, ↗tissue ALAT/ASAT/LDH, ↘tissue creatinine and proteins (except for Zn alone ↗tissue creatinine)
Ostróżka-Cieślik et al. (2020a)	Poland	Preclinical	Selenium (Se) Prolactine (Pr)	Pig kidney	Flush + SCS Biolasol 4 °C	2h before first perfusion and sampling + 48h + end-perfusion and sampling	Se: 1 μg/L Pr: 0,1 μg/L	At 2h: ↘ASAT/ALAT/proteins/urea in perfusate (except for Se alone) At 48h: ↘ASAT/ALAT/proteins/urea and ↗Na+/K+ balance in perfusate (except for Se alone)
Koo et al. (2001)	United Kingdom	Preclinical	Lec-SOD	HUVEC HDMEC	SCS Marshall's organ preservation solution 4 °C hypoxic	18/24/27h + 24h reperfusion	50 μg/mL	For both HUVEC/HDMEC: ↗cell viability at 27h, ↘ICAM and E-selectin, ↘Neutrophil adhesion
Nakagawa et al. (2002)	United Kingdom	Preclinical	Lec-SOD	Rat allogenic transplant	SCS Marshall's solution 4 °C	1/18h	50 μg/mL	For SCS18h: ↘proteinuria at W16/20/24, ↘granulocytes/leucocytes infiltration and ↘MHC I at D1 only, ↘apoptosis at D3/W24
Huang et al. (2003)	United States	Preclinical	Deferoxamine	Rat syngeneic transplant (ØWIT)	SCS UW 4 °C	18h	0,125 or 0,625 mM	↘kidney weight at postCS and D3/9, ↘F2-isoprostanes, ↗GFR and RBF, ↘RVR at D3, ↘creatininemia at D9, ↘apoptosis and tubular necrosis D3/9
Thuillier et al. (2011)	France	Preclinical	M101	LLC-PK1 Pig autotransplant	SCS UW/HTK/IGL/Celsior/RL or Perfadex 4 °C SCS UW/HTK 4 °C	24h 24h	0-10 g/L 5 g/L	↗viability, ↘apoptosis and necrosis (time and concentration dependent) ↗urine production and stabilization, ↘creatininemia peak level and ↗recovery, ↘FENa, ↘histology lesions/inflammation/fibrosis at M3 and correlated to ↘creatininemia/proteinuria at M3
Snoeijs et al. (2011)	The Netherlands	Preclinical	Propofol (P) + Cyclodextrin	LLC-PK1 Pig autotransplant (DCD WIT 45min)	SCS UW 4 °C Flush HTK + HMP	20h 23h	P: 0,1-0,5-1-5-10 μM P: 40 μmol/100g initial flush + 32 μmol/100g 1h after HMP initiation + 32 μmol/100g 1h before HMP discontinuation	↘ LDH release and ↗mitochondrial activity MTS-test (beginning for P: 1 μM) ↘ MDA at 2/10/30min, no significant ↘creatininemia/BUN, no difference on histological tubular lesions and inflammation
Mitochondrial metabolism targeted treatments								
Hauet et al. (1997)	France	Preclinical	Trimetazidine (TMZ)	Pig kidney	SCS Eurocollins/UW 4 °C	48h + 2h NMP reperfusion	1 μM	↗PFR and GFR, ↗Na reabsorption fraction (FRNa), ↘urinary TMAO, ↘Lactate in perfusate, ↗Citraturia, ↘MDA, ↘histological injuries
Hauet et al. (1998a)	France	Preclinical	Trimetazidine (TMZ)	Pig kidney	Flush + SCS UW 4 °C	48/72h + 2h NMP reperfusion	1 μM	↗PFR, ↗GFR, ↗FRNa, ↘glycosuria, ↘urinary LDH activity/TMAO/lactate/acetate, ↗citraturia, ↘tissue lactate, ↘tissue TMAO and Schiff bases (except at 4h reperfusion), ↘histological lesion score
Hauet et al. (1998b)	France	Preclinical	Trimetazidine (TMZ)	Pig kidney	Flush + SCS EC/UW 4 °C	48h + 2h NMP reperfusion	1 μM	↗PFR, ↘tissue oedema, ↘MDA, ↗ATP/Pi ratio, improved intracellular pH, (best with TMZ-UW)
Hauet et al. (1998c)	France	Preclinical	Trimetazidine (TMZ)	Pig kidney	Flush + SCS EC 4 °C	24/48h + 2h NMP reperfusion	1 μM	↗PFR, ↘kidney weight, ↗GFR, ↗FRNa, ↘glycosuria, ↗aminoacids excretion, ↘urinary LDH activity/ß-NAG/TMAO/Lacate, ↘tissue MDA, ↘histological lesion score
Goujon et al. (2000)	France	Preclinical	Trimetazidine (TMZ)	Pig autotransplant	Flush + SCS EC/UW 4 °C	48h	1 μM	↗GFR, ↘FENa, at W12: ↘tubular atrophy/interstitial fibrosis scores, ↘CD4/CD8/MCA1218+ cell infiltration, ↘VCAM staining (qualitative)
Hauet et al. (2000)	France	Preclinical	Trimetazidine (TMZ)	Pig autotransplant (ØWIT)	Flush + SCS EC/UW 4 °C	48h	1 μM	↗survival, ↗GFR, ↘urinary TMAO, ↘TMAO/creatinine and DMA/creatinine urinary ratios (best TMZ-UW), ↘tubular lesions/brush border loss, ↘T cells and macrophages infiltrate D3/14
Faure et al. (2003)	France	Preclinical	Trimetazidine (TMZ)	Pig autotransplant	Flush + SCS UW/HEH/CEL 4 °C	24/48/72h	1 μM	↗GFR (best with HEH), ↘tubular atrophy/interstitial fibrosis at W2/4/10/16 (best HEH/CEL), ↘CD4+ infiltration, ↘monocytes/macrophages infiltration (not with HEH), ↘PBR+ cells [mitochondrial transmembrane protein], ↘HLA II and VCAM-1 (qualitative)
Faure et al. (2004a)	France	Preclinical	Trimetazidine (TMZ)	Pig autotransplant (ØWIT)	SCS UW/Celsior/HEH/ECPEG 4 °C	24/48h	1 μM	↗GFR, ↘FENa (best ECPEG/HEH), ↗citraturia, ↘urinary TMAO, ↘mitochondrial alterations, ↘tubular lesions (cell detachment and dilatation), ↘CD4+ T cells and monocytes/macrophages infiltration
Faure et al. (2004b)	France	Preclinical	Trimetazidine (TMZ)	Pig autotransplant (ØWIT)	Flush + SCS UW/HEH 4 °C	24/48h	1 μM (+/- PEG +/- K+)	At W16: ↗GFR (best HEH + PEG + TMZ + low K+), ↘proteinuria, ↘CD4+ T cells and monocytes/macrophages infiltration, ↘interstitial fibrosis (qualitative)
Baumert et al. (2004)	France	Preclinical	Trimetazidine (TMZ)	Pig autotransplant	SCS UW/HEH/CEL 4 °C	24/48/72	1 μM	↗GFR, ↘FENa/FEMg, ↗citraturia, ↗succinate excretion, ↗PBR protein tubular expression, ↘tubular lesions, ↘VCAM/MHCII (semiquantitative)
Mister et al. (2002)	Italy	Preclinical	Propionyl-L-carnitine	Rat syngeneic transplant	Flush + SCS UW 4 °C	4h	1,2 mg/mL	↘creatininemia at 16h/24h but not after, ↘granulocytes infiltration at 16h
Ploetz et al. (2011)	Germany	Preclinical	Fumarate	Rat syngeneic transplant Rat allotransplant	Flush + SCS UW 4 °C	5h	5 mM	↗arterial hyperplasia M6 ↗mortality (40% vs 20%), ↗tubular atrophy/infiltration/arterial hyperplasia at M6, ↘functional outcomes
Gasotransmitters								
Lobb et al. (2012)	Canda	Preclinical	Sodium hydrogen sulfide (NaHS)	Rat syngeneic transplant (ØWIT)	Flush + SCS UW 4 °C	24h	150 μM	↗survival at D14, ↘creatininemia, ↗diuresis recovery and ↘proteinuria (comparable to Sham levels at D10), ↘glomerular/tubular necrosis, ↘apoptosis, ↘MPO/CD68+ cell infiltration, ↘IFNγ/ICAM gene expression
Lobb et al. (2015)	Canada	Preclinical	Sodium hydrogen sulfide (NaHS)	Rat allogenic transplant (ØWIT)	Flush + SCS UW 4 °C	6h	150 μM	↗survival, ↘creatininemia before D6, ↘tubular necrosis/apoptosis/Kim-1 at D1 until D6, ↘Kim-1/NGAL mRNA, ↘SerpinA3/Adamts1/Olr1/Timp1 mRNA, ↗mRNA related to cellular proliferation and IFNγ related genes
Lobb et al. (2017)	Canada	Preclinical	Sodium hydrogen sulfide (NaHS) AP39 (synthetic)	Rat allogenic transplant (ØWIT) NRK-52E rat kidney proximal tubular cell Rat syngeneic transplant (ØWIT)	Flush + SCS UW 4 °C SCS PBS 12°C hypoxic Flush + SCS UW 4 °C	24h 24h + 15min/18h/24h reperfusion 24h	NaHS: 150 μM AP39 or GYY4137 (control) AP39: 200 nM	↗survival, ↘creatininemia, ↘apoptosis D2/4, ↘necrosis ↗cell survival, ↗mitochondrial potential preservation, ↘ROS production ↗survival (70% vs 15% at D14), ↘creatininemia (comparable to Sham levels)
Juriasingani et al. (2018)	Canada	Preclinical	AP39	LLC-PK1 Pig kidney (DCD WIT 60min)	Static storage (SS) UW 10°/21°/37 °C hypoxic SCS/SSN UW 4°/21 °C	18h + 24h reperfusion 24h	5-50-500 nM-5 μM-10 μM 200 nM	↗cell viability, ↘cell apoptosis at 5-10 μM for SS 10°/21°/37° (best SSN 21°) ↘tissue necrosis for supplemented SSN 21° (SCS + AP39 not tested)
Juriasingani et al. (2022)	Canada	Preclinical	Sodium hydrogen sulfide (NaHS)	Rats syngeneic transplant (DCD WIT 30min) Pig autotransplant (DCD WIT 60min)	D-cysteine 2mg/kg intraperitoneal, 1h before graft recovery + Flush + SCS UW 4 °C Flush + HMP	18h 24h	150 μM	↗survival at D30 and ↘tubular necrosis (comparable to Sham levels) At end-HMP: ↗PFR, ↘RVR; ↘creatininemia at D1/7
Zhang et al. (2022)	Canada	Preclinical	AP39 Sodium Thiosulfate (STS)	NRK-52E rat kidney proximal tubular cell Rat syngeneic transplant (ØWIT)	Serum free media 10 °C hypoxic Flush + SCS UW 4 °C	24h + 24h reperfusion 24h	AP39: 200nM or STS: 50-150-500-1000 μM STS 150 μM	↗cell viability and ↘apoptosis (STS 150-500 μM and AP39) ↗survival, ↘creatininemia/BUN and ↗urine output with steadily improvement until D14 (comparable to Sham levels), ↘apoptosis D3/14 (comparable to Sham levels), ↘tubular necrosis at D3, ↘KIM-1/CD68/MPO at D14 (comparable to Sham levels), ↘(IFN- γ, TNF- α, IL-6, PARP, BAX, caspase-3, BID, JNK1, JNK2) gene expression at D3, ↗mitochondrial preservation
Nakao et al. (2008)	USA	Preclinical	CO	Rat syngeneic transplant	Flush + SCS UW 4 °C	24h	CO 5% 5min bubbled into UW (= 40,6 μM)	↘HO-1 mRNA expression at 6h [sign of reduced heme release by impared CYP], ↘MDA at 3h, ↘COX-2/IL6/TNFa/Egr-1 mRNA at 3h, ↘macrophages infiltration D28, ↗GFR D28, ↘proteinuria at D28, ↗median survival (>100 vs 51 days)
Yoshida et al. (2010)	United States	Preclinical	CO	Pig autotransplant (ØWIT) Pig allotransplant	Flush + SCS UW 4 °C	48h	CO 5% 5min bubbled into UW (= 40,6 μM)	↘creatininemia and BUN at D2/3, earlier urine production, ↘CD3+T cells at D14, ↘TGF-β protein, ↘fibrosis at D14 ↘IL-1β/IL-6/IL-18 mRNA and ↘MDA in early 3h post-reperfusion period allografts
Ozaki et al. (2012)	United States	Preclinical	CO	Rat syngeneic transplant (DCD WIT 40min) Discarded human kidneys	Flush + SCS UW 4 °C Flush + SCS UW 4 °C	24h 24h + 3/6/12h reperfusion	CO 5% 5min bubbled into UW (= 40,6 μM)	At WIT and SCS-end: ↘porin expression, cleaved-casp-3, cleaved-PARP, gp91phox, apoptosis ↘creatininemia peak D3 and animal death D10, ↘inflammation and tubular lesions, ↘apoptotic cell at 3h, ↘IL1β, IL6, IFNγ, iNOS at 3h Human: ↘apoptosis at 6/12h
Abe et al. (2012)	Japan	Preclinical	Hydrogenation of the solution	Rat syngeneic transplant	Flush + SCS UW 5 °C *Control: transplant with no SCS*	24/36/48h	1,32 mg/L of UW (= HRUW)	For SCS36h: ↗survival at D100 for SCS36h only; for SCS24h: ↘creatininemia/↘proteinuria/↗GFR at D90 (comparable to control levels), ↘tubular injuries and interstitial fibrosis (comparable to control levels) For SCS36h: ↘MDA at H3 (comparable to control levels), ↘apoptosis at H24 (comparable to control levels), ↘chemokine ligand 2/IL1β/iNOS mRNA and ↘iNOS proteins at H24
Nishi et al. (2021)	Japan	Preclinical	Hydrogenation of the solution	Pig allotransplant (DCD WIT 20min)	Flush + SCS ETK 4 °C	1h/4h	ETK hydrogenation 1ppm	*Descriptives analyses up to D100:* ↗survival, ↘BUN/creatininemia/syndecan-1, ↗kidney blood flow (US/CT), ↘resistance index and kidney arterial thrombosis (US)
Anticoagulants								
Sedigh et al. (2019)	Sweden	Preclinical	CHC	Pig kidney (DBD model)	HMP KPS-1	20h + 3h NMP reperfusion (with 1g exogenous creatinine addition)	50 mg/L (4h end-perfusion supplementation)	↘kidney weight, ↗exogenous creatine decline and urine production, ↘lactate, ↘intrarenal resistance (RVR), ↘mean arterial pressure with similar renal blood flow (RBF), ↘NGALu, ↘tubular injuries
Hamaoui et al. (2016)	United Kingdom	Preclinical	Thrombalexin (TLN)	Pig kidney (DCD WIT 15min) Discarded human kidney	HMP UW (+ 5h of previous SCS)	4h “stabilization” + 1,5h “treatment” [30min flush + 30min TLN + 30min washout] + 6h NMP reperfusion	2,1 μM 4,2 μM	TLN adherence to kidney microvasculature confirmed at immunochemistry, ↗RBF and PFR in NMP, ↗capillary diameter with same density, ↗red blood cell velocity, no alteration of coagulation parameters, Humans: ↗RBF and PFR, ↘lactate at microdialysis, ↘Ddimeres and fibrinogen
Tillet et al. (2015)	France	Preclinical	Fondaparinux	Pig autotransplant (DCD WIT 60min)	Flush + UW SCS 4 °C	24h	Fondaparinux 5mg IV + 10mg/L in UW Control: UFH IV + 5000UI/L in UW	No bleeding in any experiment, ↘creatininemia D3-D30, ↘tubular atrophy, leucocyte infiltration and intertitial fibrosis at D90, ↘α-SMA and Vimentin at J90, ↘IL-8, ↘activated cleaved PAR-2
Giraud et al. (2009)	France	Preclinical	Melagatran (M)	Pig autotransplant (DCD WIT 60min) Pig microvascular endothelial cells	SCS UW 4 °C 1h hypoxic chamber + SCS UW 4 °C hypoxic	24h 24h + 24h reperfusion	0,3 mg/L (+/- M: 0,3 mg/kg IV 30min before WI)	↘PNF, ↘Rantes/IL1-Rn/IL-1β/CD40L/Fas/Trail mRNA, ↘tubular necrosis/tubulitis/cell infiltration at D7 (best with associated IV Melagatran injection) ↗cell viability, ↘LOX-1/Nox2 mRNA [oxidative stress], ↘Thrombospondin/P-selectin/IL-1β/MCP-1 mRNA [endothelial cell activation]
Favreau et al. (2010)	France	Preclinical	Melagatran (M)	Pig autotransplant (DCD WIT 60min)	SCS UW 4 °C	24h	M: 0,3 mg/L (+/- M: 3 mg/kg/10min IV 30min before WI) or Heparin: 5000 IU/L (+/- Heparin: 500 IU/kg IV 10min before WI)	↗survival at M3 (M: 90% vs Heparin: 27% vs None: 0%), ↗GFR, ↘proteinuria, ↘fibrosis at M3 and its effectors (↘pSmad/Smad4/CTGF [effectors of TGF-β], ↘Smad7 [inhibitor of TGF-β], ↗tPA preservation and ↘PAI protein and mRNA), ↘Epithelial-Mesenchymal Transformation (↘αSMA/Vimentin, ↘S100A4 mRNA), ↘Nox2 and iNOS mRNA [oxydative NO production] (best M in UW+IV)
Thuillier et al. (2010)	France	Preclinical	Melagatran (M)	Pig autotransplant (DCD WIT 60min)	Flush + SCS UW 4 °C	24h	M: 0,3 mg/L (+/- M: 3 mg/kg/10min IV 30min before WI) or Heparin: 5000 IU/L (+/- Heparin: 500 IU/kg IV 10min before WI)	At M3: ↗survival (M: 90% vs Heparin: 27% vs None: 0%), ↗GFR, ↘proteinuria, ↘osmolarity ratio Urine/Plasma, ↘tubular atrophy and interstitial fibrosis, ↘CD3+ T cells and macrophages, ↘TNFα/IFNγ/IL2 mRNA, ↘IL1-R/IL10/IL17 mRNA, ↘P-selectine/C3 mRNA, ↗VEGF and ↘Thrombospondin-1/Notch4 mRNA [antiangiogenic factors] (best M in UW+IV)
Anti-apoptotic								
Jani et al. (2004)	US	Preclinical	Q-VD-OPH	Murine kidney	Flush + SCS UW 4 °C	48h	100 ug/mL	↘apoptosis (cortex and outer medulla), ↘tubular cell caspase-3 activity, ↘cleaved caspase-3, ↘caspase-2/8/9 activity, ↘brush border injury scores
Nydam et al. (2018)	US	Preclinical	Q-VD-OPH	M-1 renal tubular epithelial cells Murine syngeneic transplant (ØWIT)	SCS NaCl 4 °C SCS NaCl 4 °C	24h + 24h reperfusion 1h	5-50 μM 100 μM or DMSO	↘capase-3 dose-dependently, ↘cell death, ↘cleaved-caspase-3 activity ↘creatininemia D8, ↘apoptosis, ↘brush border injury, ↘tubular casts formation
Yang et al. (2011)	United Kindgdom	Preclinical	Caspase-3 siRNA	Pig kidney (WIT 10min)	Flush + SCS hyperosmolar citrate infused with vessels clamped 4 °C	24h + 3h NMP reperfusion (with 1g exogenous creatinine addition)	3 μg/mL	↘casp-3 proteins (precursor and active subunit), ↘casp-3+ cells at NMP3h, ↘apoptotic cells at NMP3h, ↘pH acidosis, ↗cell oxygen consumption, no effect on RBF/*ex vivo* creatinine clearance/total urine output
Yang et al. (2013)	China	Preclinical	Caspase-3 siRNA	Pig autotransplant (ØWIT)	Flush + SCS UW infused with vessels clamped 4 °C	24h	0,3 mg/40mL	After CS: ↘Casp-3 mRNA and protein levels, ↘active Casp-3 cell+, ↘apoptotic cells, ↘histological lesion score After autotransplant: ↗Casp-3 mRNA and protein levels, ↗active Casp-3 cell+, ↗apoptotic cells, ↗MPO+ cells infiltration, ↗histological lesion score, no effect on creatininemia and BUN at 48h
Yang et al. (2014)	China	Preclinical	Caspase-3 siRNA (Stabilized)	Pig autotransplant (ØWIT)	Flush + SCS UW infused with vessels clamped 4 °C	24h	0,3 mg/40mL + 0,9 mg IV before autotransplant	↘Casp-3 mRNA, ↘Casp-3 protein levels, ↘apoptotic cells, ↘PNN, ↘IL-1β/IL-6/NF-κB/IFN-α/IFN-β/IFN-γ/IRF3/IRF7/IFIT1 mRNA at D0/D14, ↘blood peripheral proinflammatory cytokines D6-D14, ↗IL4/ IL10 at D4-D8, ↘creatininemia and BUN at D11-D14, ↘tubulointertitial lesions and extracellular matrix deposition at D14, ↘HMGB1 protein levels at D0/D14
Anti-inflammatory								
Ćelić et al. (2018)	Croatia	Preclinical	rhBMP-7	Rat kidney (ØWIT)	SCS NaCl/UW 4 °C	6/12/24h	10 R/kg	↘brush border loss, ↗BMP-7 mRNA and proteins in tubular cells and podocytes, ↘TGF-β, ↗Smad 1/5/8, ↘Smad 2/3, ↗E-cadherin expression, ↘α-SMA, ↗Hsp70, ↘activated caspase-3, ↗HIF-1α
Doucet et al. (2008)	France	Preclinical	FR167653	Pig autotransplant (DCD WIT 60min)	Flush + SCS UW 4 °C	24h	60 mg/L (+/- 1mg/kg IV before WI and autotransplant)	↗survival, ↗GFR, ↘proteinuria at M3, ↘TNFα and IL1β at H1H3, ↗histology preservation at D1, ↗tubule regeneration at D1/7, ↗mitochondrial preservation at D1, ↗N-cadherin preservation in proximal tubule, ↗HIF1α and VEGF/VEGF-R in proximal tubular cells, ↘fibrosis and mesenchymal transition at M3 (α-SMA, vimentin, CD3+ and ED1 interstitial infiltration)
Lewis et al. (2008)	United States	Preclinical	C5aRA	Murine syngeneic transplant (DCD WIT 30min)	Flush + SCS UW 4 °C	2h	1 μM	↗mice survival at D2, ↘kidney tissue damage at 72h, ↘apoptotic tubular cells, ↘C5aR protein and mRNA tubular cell expression, ↘TNFα and MIP-2/CXCL2 expression
Ostróżka-Cieślik et al. (2020b)	Poland	Preclinical	LH + Vitamin C	Pig kidney	Flush + SCS Biolasol 4 °C	2h before first perfusion and sampling + 24h end-perfusion and sampling	0,01-0,1-1 μg/L	At end-perfusion and 30min sampling: ↘AST/ALT, ↘urea, ↗Na/K ratio preservation (best 0,01 μg/L)
Singh et al. (2013)	United States	Preclinical	Zinc-N-acetylcystein (ZnNAC)	NRK-52E rat kidney proximal tubular cell Rat kidney	SCS UW 0 °C In situ Flush + SCS UW 0 °C	24h + 18h reperfusion 24h	0-1-3-10-30 mM 0,3-1-3-10-30 mM	↗cell survival at 3-10 mM ↘apoptosis at 1-30 mM, ↘cleaved caspase-3 and endonuclease G
McAnulty et al. (2002)	United States	Preclinical	TFS (Growth Factors)	Dog autotransplant (ØWIT)	Flush + SCS UW 4 °C	72-96h (control) or 96-146h (test)	Bovine Neutrophil Peptide-1 1 mg/L + Substance P 2,5 mg/L + NGF-β 20 μ/L + IGF-1 10 μg/L + EGF 10 μg/L	↘creatininemia peak D2/12, ↘creatininemia recovery duration
Kwon et al. (2007)	United States	Preclinical	TFS (Growth Factors)	Primary canine kidney tubular cells Madin-Darby canine kidney cells HUVEC	SCS UW 4 °C	96h + 1/4/6/24h reperfusion	Bovine Neutrophil Peptide-1 1 mg/L + Substance P 2,5 mg/L + NGF-β 20 μ/L + IGF-1 10 μg/L	↗membrane potential preservation (only for HUVEC at 4h reperfusion), ↘activated caspase-3
Petrinec et al. (1996)	United States	Preclinical	IGF-1	Dog autotransplant (ØWIT)	Flush + SCS Eurocolins 4 °C	24h	0,1 μM	↘creatininemia at D6, ↘BUN at D5/6, ↗inulin clearance at D6

### 3.2 Insights from clinical studies

The main clinical study was conducted by Nicholson et al. (1996) and tested the calcium channel blocker nicardipine, which was supposed to limit IRI, added to Euro-Collins solution used in SCS to evaluate its impact on DGF (Nicholson et al., 1996). A total of 65 consecutive dead brain donors (DBDs) were enrolled, resulting in 127 kidney transplantations, including 62 with nicardipine adjunction; however, no difference in DGF was found. More recently, phase I clinical trials investigating the oxygen transporter and antioxidative agent M101 and the anticoagulant Corline Heparin Conjugate (CHC, also known as Renaparin) have also been reported (Le Meur et al., 2020; Sedigh et al., 2022). No significant difference in adverse events was found for either drug in the early to mid-term period, with mitigated results on kidney function; however, the study designs were not suitable for drawing definitive conclusions. Compared to the few clinical studies available to date, numerous preclinical studies have investigated a wide range of pharmacological agents, including antioxidants, metabolism-targeted treatments, gaseous agents, anticoagulants, anti-apoptotic agents, and anti-inflammatory agents.

### 3.3 Pharmacological agents from preclinical studies

#### 3.3.1 Antioxidants

##### 3.3.1.1 Flavonoids

Flavonoids belong to a family of natural polyphenolic compounds widely recognized as antioxidants. In two studies involving porcine proximal tubular cell models, researchers have found that adding certain flavonoids to preservation solutions resulted in preserving cell morphology and integrity, reducing ROS formation, and decreasing ATP depletion (Ahlenstiel et al., 2006; Karhumäki et al., 2007). One study focused on tanshinone IIA supplementation during *ex vivo* rat kidney cold storage in Celsior and found concordant results with an upregulation of superoxide dismutase (SOD) and a decrease in pro-oxidant and pro-apoptotic factors (Zhang et al., 2012). So far, resveratrol is regarded as one of the most studied flavonoid since two studies have confirmed its protective effects in DCD porcine autotransplantation models for SCS and HMP supplementation; however, both studies tested two different formulations of resveratrol (ADD10 and Vectisol) (Cassim et al., 2022; Soussi et al., 2019). Functional benefit was confirmed by improved tubular function and reduced creatininemia and fibrosis in the post-transplant setting up to 3 months.

##### 3.3.1.2 Direct scavengers

Apart from flavonoids, many other compounds have been screened for their ability to scavenge ROS. In four studies involving tubular and endothelial cell models, lazaroids were shown to prevent lipid peroxidation and reduced oxidative stress, preserving cell structure and limiting apoptosis (Killinger et al., 1992; Salahudeen et al., 1999; Salahudeen et al., 2000; Salahudeen et al., 2001). Five studies were conducted on *ex vivo* rat, rabbit, dog, and pig kidneys. Of these, two focused on mitoquinone and showed better preservation of mitochondrial respiration chain activity after 4 h–24 h of cold storage, with decreased superoxide and peroxynitrite levels. These effects resulted in less apoptosis and an improvement in histological lesion scores after 48 h of cold ischemia (Mitchell et al., 2011; Parajuli et al., 2012). One study on PrC-210 added to the UW solution resulted in reduced caspase activity and acute tubular necrosis at 30 h of cold storage (Verhoven et al., 2020). Only a slight benefit of Trolox, a water-soluble vitamin E analog, has been reported on MDA levels and Schiff bases in two studies (McAnulty and Huang, 1997; McAnulty and Huang, 1996). Furthermore, five studies conducted on *ex vivo* rat, dog, and pig kidney models focused on vitamin C, zinc, and selenium for preservation fluid supplementation, respectively; however, a negative impact on cytolysis and oxidative stress markers was recorded, although data were limited (McAnulty and Huang, 1997; McAnulty and Huang, 1996; Ostróżka-Cieślik et al., 2018; Ostróżka-Cieślik et al., 2021; Ostróżka-Cieślik et al., 2020a). After investigations showing improved cell viability and decreased neutrophil adhesion in human endothelial cells, Lec-SOD was directly introduced into a rat kidney allotransplantation model (Koo et al., 2001). Reduced apoptosis and immune cell infiltration up to 24 weeks, along with decreased proteinuria, were found (Nakagawa et al., 2002). Similarly, although the iron chelator deferoxamine showed promise for lipid peroxide reduction in four studies on kidney tubular cells (Salahudeen et al., 1999; Salahudeen et al., 2000; Salahudeen et al., 2001; McAnulty and Huang, 1997), introduction into a syngeneic rat kidney transplant model found improvement in tubular necrosis and glomerular filtration rate during the first 10 days (Huang et al., 2003). Two studies on porcine autotransplantation investigated propofol and M101 for fluid supplementation. Due to its intrinsic SOD activity, M101 supplementation has demonstrated benefit in improving kidney functional and histological outcomes up to 3 months in 24-h static cold-stored kidneys (Thuillier et al., 2011). However, after 23 h of hypothermic perfusion of DCD kidneys with propofol, no improvement in kidney function or histology was found, despite previous benefit in isolated porcine tubular cells (Snoeijs et al., 2011).

#### 3.3.2 Mitochondrial metabolism-targeted treatments

Targeting cell metabolism is another strategy to enhance organ preservation through conservation of ATP stocks. Ten studies investigated trimetazidine in porcine models. Trimetazidine supplementation of UW or Euro-Collins during 24–72 h of SCS of porcine kidneys demonstrated improved perfusion parameters during 2 h of normothermic reperfusion. In addition, improvements in glomerular and tubular function [*ex vivo* glomerular filtration rate (GFR) and reabsorption fraction of sodium] and oxidative and kidney injury markers in the perfusate, urine, or tissue [lactate, LDH, and trimethylamine N-oxide (TMAO)] were observed, along with reduction in histological lesions (Hauet et al., 1997; Hauet et al., 1998a; Hauet et al., 1998b; Hauet et al., 1998c). These results were confirmed in porcine autotransplantation studies with up to 4 months of follow-up and using various preservation solutions (Goujon et al., 2000; Hauet et al., 2000; Faure et al., 2003; Faure et al., 2004a; Faure et al., 2004b; Baumert et al., 2004). One study on rat syngeneic transplantations after 4 h of cold storage in UW supplemented with propionyl-L-carnitine has shown a significant decrease in creatininemia and granulocyte infiltration in the first 24 h (Mister et al., 2002). Lastly, one rat kidney transplantation study investigated fumarate, but it was associated with increased mortality and histological lesions, with worst kidney functional outcomes indicated by creatininemia and proteinuria up to 6 months (Ploetz et al., 2011).

#### 3.3.3 Gasotransmitters

Inspired by the physiological role of endogenous gaseous signaling molecules, gaseous-derived pharmacologies have attracted interest. In five studies investigating different forms of hydrogen sulfide donors from cellular to rat and porcine transplantation models, sodium hydrogen sulfide (NaHS), the synthetic mitochondria-targeted AP39, and sodium thiosulfate (STS) have demonstrated benefits in improving cold preservation of kidneys. Using a 24-h SCS in the UW preservation method, animal survival was increased up to 1 month, with reduced creatininemia and acute tubular necrosis, confirmed by reduction in the expression of Kim-1 and NGAL tubular injury markers during the first 2 weeks (Lobb et al., 2012; Lobb et al., 2015; Lobb et al., 2017; Juriasingani et al., 2018; Juriasingani et al., 2022; Zhang et al., 2022). Decreased levels of ROS production, pro-inflammatory cytokines, and granulocyte infiltration were also observed at day 14 (Lobb et al., 2012; Lobb et al., 2017; Zhang et al., 2022). Notably, only one study investigated hypothermic perfusion using a 24-h cold preservation period for DCD porcine kidneys perfused with NaHS-supplemented UW and reported short-term benefits in perfusion flow, renal vascular resistance, and first-week creatininemia (Juriasingani et al., 2022).

Carbon monoxide (CO) has also been tested in three preclinical studies. After dissolving CO into UW to reach a reproducible concentration of 40.6 μM, the effect of CO was tested in rat and porcine kidney transplantations using a 24–48-h SCS method (Nakao et al., 2008; Yoshida et al., 2010; Ozaki et al., 2012). A downregulation of HO-1 cytoprotective signaling pathway mRNA expression was observed, indirectly suggesting reduced free heme release induced by IRI. A reduction in early oxidative markers, pro-inflammatory cytokines, and apoptosis was found. Mid-term outcomes were also improved, as evidenced by GFR and proteinuria at day 28, immune cell infiltrate and fibrosis at day 14, and animal survival up to 100 days. Lastly, a histological study was performed on discarded *ex vivo* human kidneys after 24 h of SCS in UW and following 3–12 h of normothermic reperfusion and revealed a concordant decrease in TUNEL apoptosis after 6 and 12 h of reperfusion (Ozaki et al., 2012).

The effect of preservation fluid supplemented with dissolved hydrogen was investigated in two studies. Using a rat syngeneic kidney transplantation model, glomerular function, tubular injury, and interstitial fibrosis showed significant improvement at 3 months following a 24-h cold storage period (Abe et al., 2012). Extending the cold storage period up to 36 h resulted in a better animal survival rate at day 100, with improved oxidative, inflammatory, and apoptosis parameters. Although descriptive, animal survival, kidney function, and *in vivo* ultrasonography kidney blood perfusion up to 100 days seemed enhanced in a DCD porcine allotransplant model during short-term cold storage (Nishi et al., 2021).

#### 3.3.4 Anticoagulants

The use of heparin-derived molecules has been investigated in several porcine kidney models. Two studies investigated thrombalexin and CHC. After HMP and normothermic reperfusion, both have shown better perfusion parameters during normothermic machine perfusion (NMP), and CHC also demonstrated improved glomerular function and tubular injuries (Sedigh et al., 2019; Hamaoui et al., 2016). Fondaparinux and melagatran were investigated in four studies involving DCD pig autotransplant models after 1 h warm ischemia time (WIT). Of note, supplementation of the preservation solution, with or without an intravenous injection, at the time of transplantation, was studied. Fondaparinux improved creatinine clearance at 1 month and reduced kidney histological scarring and epithelial–mesenchymal transformation markers at 3 months (Tillet et al., 2015). In addition to demonstrating increased viability with lower oxidative stress and activation in a porcine endothelial cell model, melagatran improved glomerular and tubular function up to 3 months, with reduced pro-inflammatory gene expression and epithelial–mesenchymal transformation markers (Giraud et al., 2009; Favreau et al., 2010; Thuillier et al., 2010). Histological findings were consistent with reduced tubular atrophy, cell infiltration, and fibrosis. It is worth noting that no difference in bleeding was observed in any of the *in vivo* studies.

#### 3.3.5 Anti-apoptotic agents

Direct inhibition of apoptosis by targeting caspases has been tested to improve kidney graft quality. Pan-caspase inhibition by Q-VD-OPh in two studies and exclusive caspase-3 inhibition by small interfering RNA (siRNA) in three studies have been reported. A reduction in caspase activity and cell death was observed, after Q-VD-OPh supplementation in murine kidney tubular cells, resulting in lower creatininemia, apoptosis, and tubular lesions at 1 week after kidney syngeneic transplantation in mice (Jani et al., 2004; Nydam et al., 2018). Yang et al. reported the use of caspase-3 siRNA in normothermic reperfusion pig kidney and autotransplant models after supplementation for SCS. Although caspase-3 expression, apoptosis, and histological lesions were decreased after 24 h of cold storage and 3 h of normothermic reperfusion, worst outcomes were initially observed after autotransplantation (Yang et al., 2011; Yang et al., 2013). A poor serum stability of the siRNA and systemic compensative responses led the authors to modify the siRNA and associate systemic injection of siRNA at the time of autotransplantation. Up to 2 weeks, the efficacy of the new siRNA was confirmed, which was supported by an improved anti-inflammatory systemic cytokine expression profile, enhanced glomerular function, and reduced tubulointertitial injuries (Yang et al., 2014).

#### 3.3.6 Anti-inflammatory agents

FR167653 is a potent anti-inflammatory drug that inhibits the MAPK–p38 signaling pathway. rhBMP-7 impairs fibrosis development by inhibiting epithelial–mesenchymal transition. Although the use of UW supplemented with FR167653 in a DCD porcine kidney autotransplantation after 24 h of SCS showed an improvement in glomerular filtration and proteinuria, with reduced inflammatory cytokine expression, both treatments demonstrated better tubular cell preservation, with less mesenchymal transition up to 3 months, along with increased HIF-1α expression levels (Ćelić et al., 2018; Doucet et al., 2008). Blockage of the complement system by the C5aR antagonist A8 has also been described in a murine syngeneic transplant study, resulting in improved animal survival at day 2, with better kidney tissue preservation and decreased inflammatory cytokine release (Lewis et al., 2008). Data on LH, prolactin, zinc-N-acetylcysteine, and growth factors were scarce, with only eight cellular and *ex vivo* kidney studies focused on their ability to promote cell viability and nephroprotection (Ostróżka-Cieślik et al., 2018; Ostróżka-Cieślik et al., 2021; Ostróżka-Cieślik et al., 2020a; Ostróżka-Cieślik et al., 2020b; Singh et al., 2013; McAnulty et al., 2002; Kwon et al., 2007; Petrinec et al., 1996).

## 4 Discussion

### 4.1 Current state of investigations into pharmacological agents

The aim of this review was to gather and summarize the investigations of all the tested pharmacological agents used as treatment additives to the preservation fluid to improve hypothermic kidney graft preservation. Sixty-seven studies were included, three of which were clinical trials, including one negative phase III and two encouraging phase I studies. Other clinical trials on preservation solution pharmacology supplementation have been registered, without any publication so far. Although some may have provided negative outcomes and were not published, hopeful recent trials are ongoing and summarized in Table 2. Three of these clinically investigated molecules previously identified in preclinical research have been included herein. The results of the M101 phase III clinical trial are yet to be published, whereas CHC phase II and STS phase I trials are still ongoing (NCT04181710, OXYOP2^1^; CTIS2022-501389-23-02, RENAPAIR02^2^; NCT04292184^3^). Among the remaining pharmacological agents identified in preclinical studies, the vast majority showed a benefit in IRI prevention, and the most promising agents evaluated in porcine transplant studies were trimetazidine (six studies), melagatran (three studies), and ADD-10 and Vectisol (resveratrol), CO, hydrogen, fondaparinux, FR167653, and the stabilized caspase-3 siRNA (one study each). Despite the need for confirmatory results due to limited number of individuals studied, lack of a DCD model with HMP preservation, and short-term outcome evaluations in most studies, we believe that translation into clinical investigations of melagatran and FR167653 should be encouraged.

**TABLE 2:** Registered clinical investigations on pharmacological agents for hypothermic kidney preservation fluid supplementation.

Main ID, name, author	Last date of update	Country	Clinical phase	Status	Enrolment (n)	Pharmacology	Individuals studied	Preservation condition	Pharmacology dose	Outcome
NCT04181710, OXYOP2^1^	2023	France	P3	Completed	490	M101	DCD DBD	French standard of care	1 g/L	DGF, PNF, graft/patient survival, GFR at Y1, interstitial fibrosis at M3, rejection at Y1, QoL, and safety
CTIS2022-501389-23-02, RENAPAIR02^2^	2024	Germany, Austria, and United Kingdom	P2	Ongoing	Not yet recruiting	CHC—Renaparin	Deceased donors	SCS	100 mg 90 min before transplantation	Pharmacology and toxicity
NCT04292184^3^	2023	Canada	P1	Completed	18	STS	DCD	HMP UW	100 mL of 500 μM	Slow graft function, creatinine, eGFR, PBR, and Kim-1/NGAL at D7
NCT01285375	2011	Finland	P1	Unknown	20	Curcumin–cyclodextrin complex	Unknown	Flush + SCS UW	2 mL of 12 mg/mL	Safety, DGF at D7, PNF, acute rejection, graft and patient survival at Y1, and GFR at M1M3

### 4.2 Pathophysiological mechanisms and scientific issues

**FIGURE 2:**
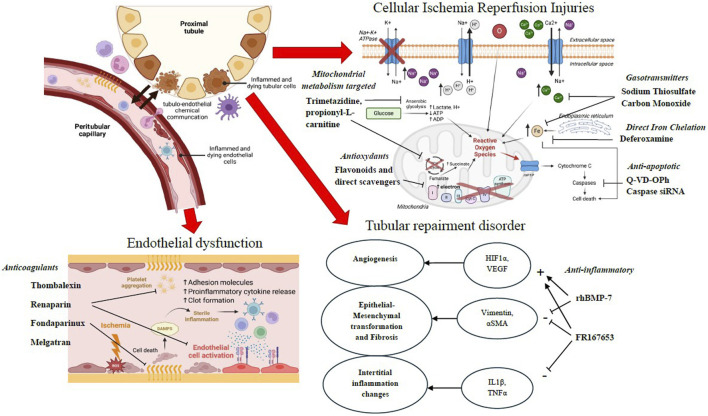
Pathophysiological mechanisms of the reported pharmacological treatment additives.

Interestingly, the complexity of IRI pathophysiology led to the investigation of a wide variety of pharmacological agents, as summarized in Figure 2. As a cornerstone of IRI, oxidative stress and ROS formation have been a major target to improve kidney preservation during cold storage (Ostróżka-Cieślik, 2022). Many antioxidants have been investigated so far, including flavonoids, acting by direct scavenging or upregulation of endogenous enzymes (Cheng et al., 2024). They also demonstrated other promising properties for fighting against IRI, such as antithrombotic and anti-inflammatory properties (Ahlenstiel et al., 2003; Rauf et al., 2017). However, their difficulty to cross the mitochondrial membrane, where ROS are generated, have questioned their potential, leading to the development of modified mitochondria-targeted antioxidants, such as mitoquinone and PrC-210, whose clinical use remains largely investigatory (Ogurlu et al., 2024). Free iron is a major contributor to oxidative stress, which promotes ROS and lipid peroxide formation, leading to cytochrome degradation, free heme deposition, and ferroptosis (Huang and Salahudeen, 2002; Pefanis et al., 2019). This paved the way for the use of iron chelator deferoxamine and carbon monoxide, the latter being endogenously produced by heme oxygenases (HOs) through catalysis of heme (Nakao et al., 2008). However, the most promising results have been observed with hydrogen sulfide, along with its biological effector known as thiosulfate (Zhang et al., 2021; Wu et al., 2025). Thiosulfate presents antioxidant effects, by direct scavenging and increasing endogenous enzyme activity, along with iron- and calcium-chelating and vasodilatory properties. As previously mentioned, the beneficial effects of sulfate donor molecules on kidney graft preservation demonstrated in six of the included studies have led to an ongoing clinical trial (). Another strategy to improve organ preservation is to optimize cell metabolism and preserve ATP stocks. Although its mechanism of action is not fully understood, trimetazidine has been thoroughly investigated in many porcine kidney autotransplant models due to its protecting effects on myocardial ischemia (Dézsi, 2016). Shifting cell metabolism from lipid to glucose oxidation to optimize limited oxygen supply in early ischemia and managing cell acidosis are the main hypotheses of action (Morin et al., 2001). To the best of our knowledge, despite yielding interesting mid-term results, no clinical study has been initiated so far. Contrary to trimetazidine, propionyl-L-carnitine promotes lipid β-oxidation, demonstrating positive but still limited results. Although surprising, its effect may be explained through its ability to promote endogenous antioxidant enzyme activity, to scavenge superoxide and hydrogen peroxide, and to chelate iron (Ostróżka-Cieślik, 2022). Alterations in kidney vasculature are also a source of concern since endothelium impairment can lead to thrombosis or trigger immune cell recruitment, initiating alloimmune response and potential chronic graft injury. The use of heparins has been investigated to cope with the loss of the protective glycocalyx and endothelial cell injuries related to IRI. Thombalexin and CHC have been modified to target and bind the endothelium or cover the exposed extracellular matrix, preventing from thrombosis initiation and leukocyte adhesion (Ma et al., 2024). Although the aforementioned effects of thrombalexin have been confirmed in discarded human kidneys during NMP, a phase II clinical trial involving CHC is currently ongoing (Hamaoui et al., 2016) CTIS2022-501389-23-02, RENAPAIR02^2^.

However, the complexity of IRI pathophysiology has presented significant challenges, and some studies with negative outcomes have also been reported. Based on a previous positive study in a rat model of ischemic heart, fumarate was investigated for its potential to improve kidney preservation; however, it led to increased graft injuries (Ploetz et al., 2011). This phenomenon may be due to succinate accumulation, which in turn triggers a burst of ROS during reperfusion (Chouchani et al., 2014). Another example is the use of vitamin C. Although having a strong preventing and scavenging effect on ROS, vitamin C is also known to act as a prooxidant in the presence of free transition metals (Gęgotek and Skrzydlewska, 2022). As free iron is released during IRI (Huang and Salahudeen, 2002), this characteristic may explain why vitamin C was associated with no benefit or even increased oxidative stress in the included studies, whether used alone or in combination with deferoxamine (McAnulty and Huang, 1997; McAnulty and Huang, 1996; Ostróżka-Cieślik et al., 2018). Surprisingly, only very few preclinical reports were negative among those screened, while pharmacological agents that have been clinically investigated were even scarcer. An evident publication bias that limits the spreading of negative experimental reports impairs the progress of this pharmacology-based strategy for improved organ preservation (Saat et al., 2016).

### 4.3 Study limitations and overview of pharmacological supplementation strategies

Beyond the preclinical design of most of the studies which still limits the clinical value of the tested pharmacological agents, the extreme heterogeneity of methodology used must be highlighted. Indeed, differences in the individuals studied (type of cell, type of animal, DBD, or DCD), preservation solutions used (UW, HTK, EC, or Celsior), hypothermic preservation settings (temperature, static or machine perfusion assisted, machine device, or duration of preservation), and modalities of preservation solution addition impair comparability of the investigations and translation to clinical studies (Table 1). Notably, administration of the drug to the kidney during SCS remains unclear in some studies. It was not always specified whether the kidney was flushed with the augmented solution, raising questions about the delivery of the pharmacological agents if the organ was only immersed in the supplemented solution without being rinsed. Thus, caution must still be exercised to the interpretation of these outcomes. Moreover, the opening of the kidney capillaries during machine perfusion and the recurrent circulation of the drug added to the perfusion fluid, repeatedly pumped into the kidney, advocate for the use of machine perfusion to optimize pharmacology delivery (Schutter et al., 2021). Fitting to the current trend to widen clinical indications of hypothermic and normothermic perfusion, pharmacologic adjunct evaluation should now incorporate machine perfusion (Malinoski et al., 2023; Hosgood et al., 2023).

Different treatment modalities to better preserve the kidney can be used. This review focused on *ex vivo* kidney graft treatment through supplementation of the preservation solution which we believe to be the best and safest strategy. The treatment of the donor involves ethical issues, and recipients face the burden of potential side effects. Moreover, the post-reperfusion treatment strategy is rather inopportune as IRI has already been initiated and it can be challenging to mitigate. A higher and risky systemic posology to reach an effective dose into the graft could also be necessary. Direct targeting of the kidney graft during the *ex vivo* preservation period can overcome all these issues: a minimal effective dose without any exposure of the recipient before the initiation of IRI for a better prevention. The timing of treatment addition can also be chosen, from the beginning of the preservation period to few hours before the transplant surgery. Yet, the hypothermic preservation method can be criticized since the metabolism of the pharmacological agent is limited. Indeed, the landscape of kidney preservation modalities is evolving, although these approaches have not yet been integrated into current clinical practices. All investigations conducted in hypothermic settings presented herein are not applicable to studies involving different temperatures or oxygenated preservation methods, and subnormothermic or normothermic perfusion, even with controlled rewarming techniques, will significantly alter the future of the *ex vivo* pharmacological strategy (Ogurlu et al., 2024; Resch et al., 2020; Hosgood et al., 2015). For instance, we can anticipate that each additive or combination would have a preferred usage and timing of adjunction, depending on their mechanism of action to fit the occurring stage of IRI pathophysiology. Thus, antioxidants and metabolic stabilizers could be mostly suitable for oxygenated HMP to overcome electron and prooxidant accumulation, while control rewarming would benefit from anti-apoptotic agents and gasotransmitters (STS) to manage oxidative stress, chelate ions, promote cell survival, and favor capillary vasodilation, before using anticoagulants and anti-inflammatory agents during NMP to optimize capillary density and prevent from kidney scarring and immune cell trigger. Moreover, a recent review has also highlighted differences in kidney graft lesion profiles depending on their DBD or DCD origin, with the former exhibiting stronger inflammatory responses whereas the latter inducing predominant cell death lesions (Yang et al., 2024). Additive choices could also depend on the donor profile, favoring anti-inflammatory agents for DBD and anti-apoptotic agents for DCD. However, since hypothermic preservation is the gold standard to date, the clinical impact of the discovery of a treatment or a combination of drugs under the usual preservation conditions would be tremendous.

### 4.4 Limitations of the review and investigation perspectives

This review provides a comprehensive overview of the pharmacological agents investigated for supplementation of the hypothermic kidney graft preservation fluid, although the number of databases screened was limited. We believe that the wide spectrum of the searching terms and the extensive reading of the articles’ reference list must have covered the essential available literature. Furthermore, although other molecules have been tested in the general setting of kidney IRI or in kidney transplantation for donor/recipient treatment, the scope of this review was to focus on kidney graft hypothermic fluid supplementation only since no other review gathered the up-to-date research on this specific topic, to our knowledge. Yet, although many interesting preclinical findings have been highlighted, external validity of the pharmacological addition strategy is still limited. Beyond the aforementioned heterogeneity of models and protocols, it should not be overlooked that extrapolation of improvements observed in preclinical settings with any pharmacological intervention suffers from the intrinsic inaccuracy of models, voluntarily designed to induce sufficient injuries to maximize drug benefit, which is far from the clinical practice optimization spirit of achieving the best patient outcomes. Consequently, benefits of using certain additives become less obvious when best practices such as minimizing ischemia time and properly utilizing machine perfusion techniques are already being implemented. We believe that investigations in this field would benefit from fundamental research to better understand IRI pathophysiology and from standardization of hypothermic preservation settings, along with preclinical models, such as pig autotransplantation with 1-h WIT induced by kidney arterial clamping for DCD models. As previously mentioned, new preservation technologies such as normothermic perfusion, controlled rewarming, and oxygenated hypothermic perfusion should also be implemented to fit the future standards of preservation, even while traditional methods such as SCS play crucial roles for economic reasons. Publication of negative studies would also be of great interest and should be encouraged, implying modifications of scientific journal policy standards. By improving kidney preservation with hope to enhance long-term graft survival and to decrease the overall cost of chronic kidney disease, economic support from public institutions or the industry would be a win–win opportunity, aiming at the benefit of patients. Investigations should continue, with the aim of eventually translating findings into clinical practice.

## 5 Conclusion

In the era of extended kidney graft transplantations, research on improving graft preservation to manage IRI is of particular importance. Although not new, pharmacology supplementation of the preservation solution remains a promising strategy for its safety and ease of application in the clinic. This review provided an overview of all the investigated treatments in the hypothermic setting, with numerous positive preclinical results. However, the complexity of IRI pathophysiology and the lack of negative data publications impair the translation into clinical studies. Although research into pharmacological improvement in kidney graft preservation should be encouraged and would benefit from standardization and the implementation of new preservation technologies, the results of some hopeful ongoing clinical investigations are yet to be published.

## Footnotes

1 Available at: https://clinicaltrials.gov/study/NCT04181710.

2 Available at: https://trialsearch.who.int/Trial2.aspx?TrialID=CTIS2022-501389-23-02.

3 Available at: https://clinicaltrials.gov/study/NCT04292184.
